# HIV self-testing practice and associated factors among governmental medical college students in Addis Ababa, Ethiopia, 2024

**DOI:** 10.1186/s12879-026-13889-0

**Published:** 2026-06-26

**Authors:** Yonas Wondie, Meaza Damtie, Ayenew Genet Kebede, Aragaw Egziabherfenta Tadele, Asrat Yazew, Alemnew Getachew Haile, Lalem Tilahun Kebede, Eyasu Bamlaku Golla, Awoke Minwuyelet

**Affiliations:** 1 Department of Nursing, College of Medicine and Health Sciences, Injibara University, Injibara, Ethiopia; 2 Department of Public Health, Yanet College, Addis Ababa, Ethiopia; 3 Department of Health Promotion and Behavioral Science, Motta Town Health Office, Motta, Ethiopia; 4 Department of Pediatrics and Child Health Nursing, College of Medicine and Health Sciences, Injibara University, Injibara, Ethiopia; 5 Department of Public Health, College of Health Science, Oda Bultum University, Chiro, Ethiopia; 6 Department of Medical Laboratory, Bichena Primary Hospital, Bichena, Ethiopia

**Keywords:** Addis Ababa, Ethiopia, HIV self-test, Medical student, 2024

## Abstract

**Background:**

Despite global health progress, HIV/AIDS continues to challenge many countries. HIV self-testing is a key strategy to improve testing and early detection. Nevertheless, medical students, who are at risk from both occupational and non-occupational exposure, are often overlooked, leaving many unaware of their HIV status and with limited access to counseling and testing services.

**Objectives:**

This study aimed to assess HIV Self-Testing Practice and Associated Factors among Governmental Medical College Students in Addis Ababa, Ethiopia, 2024.

**Methods:**

An institution-based cross-sectional study was conducted among 375 medical students at a government college in Addis Ababa. Data were collected via a self-administered questionnaire and analyzed using SPSS version 26. Binary logistic regression was performed, and variables with *p* < 0.25 were included in the multivariable model. Associations with *p* < 0.05 and 95% confidence intervals were considered statistically significant.

**Result:**

A total of 375 health science students participated (100% response rate). The prevalence of oral HIV self-testing was 19.2% (*n* = 72). Factors positively associated with HIVST practice included participation in HIV programs (AOR = 3.27; CI: 1.30–8.21), being male (AOR = 1.74; CI: 1.02–2.82), recent HIV risk exposure (AOR = 2.84; CI: 1.35–5.98), and a history of sexually transmitted infection (AOR = 3.18; CI: 1.42–7.12).

**Conclusion:**

The prevalence of oral HIV self-testing among students at the governmental medical college was 19.2%. Participation in HIV programs, recent HIV risk exposure, male sex, and a history of sexually transmitted infections were significantly associated with HIVST practice.

**Trial registration:**

Not applicable.

## Introduction

Human Immunodeficiency Virus (HIV) remains a major global public health problem despite advances in prevention, diagnosis, and treatment [1]. In 2023, an estimated 39.9 million people globally were living with HIV, and about 86% of them knew their HIV status [2]. In sub-Saharan Africa, HIV testing gaps remain substantial, particularly among men, with reports indicating that a considerable proportion of men living with HIV are still unaware of their HIV status [3].

Ethiopia has adopted the UNAIDS 95-95-95 targets, which aim for 95% of people living with HIV to know their HIV status, 95% of those diagnosed to receive antiretroviral therapy (ART), and 95% of individuals on ART to achieve viral suppression [4, 5]. Recent reports indicate that Ethiopia has achieved approximately 90%, 94%, and 96% of the three targets, respectively, demonstrating substantial national progress toward epidemic control [6]. Despite this progress, a proportion of people living with HIV remain unaware of their status, particularly among populations with lower uptake of conventional HIV testing services, including men, young people, and individuals in underserved settings. Barriers such as stigma, concerns about confidentiality, low perceived risk, and limited access to health facilities continue to affect HIV testing uptake. Therefore, targeted and innovative strategies such as HIV self-testing (HIVST), community-based distribution, and peer or network-based approaches may help reach the remaining undiagnosed populations and support achievement of the national HIV targets [7].

In Ethiopia, HIV remains a major public health concern, with an estimated 610,000 people living with HIV in 2020 [8]. Addis Ababa has a relatively higher adult prevalence of HIV (3.58%) compared to the national average, and there are disparities in reaching the optimal level of diagnosis coverage [9]. Stigma, fear of discrimination, confidentiality concerns, and inaccessibility of limited services are the major challenges to conventional HIV testing services [10, 11]. These challenges are more common among young people, including university students, who tend to shun facility-based testing due to social judgment and privacy concerns [12, 13].

HIV self-testing (HIVST) has been recognized as a complementary approach to enhance HIV-testing uptake [14]. HIVST enables a person to take their own sample, test, and interpret the results in private, thus improving autonomy and confidentiality [15]. Evidence from African contexts shows that HIVST enhances testing use, particularly among groups who are less likely to access conventional testing services [16, 17]. Oral fluid–based HIV self-test kits are often preferred because they are non-invasive, convenient, and easy to use, although acceptability can vary depending on individual preferences and context [18, 19]. The advantages of HIVST include privacy, flexibility in testing sites, reduced stigma, and the ability to test partners [20]. However, challenges such as low awareness, misinterpretation of results, cost, and poor regulatory frameworks may hinder its adoption [21, 22].

Medical students are an important group for HIV prevention because they are typically in a sexually active age range where studies among university students in Africa have reported behaviors such as inconsistent condom use and multiple sexual partners [23, 24]. Even though they are expected to have strong knowledge about HIV, evidence still shows that HIV testing uptake among health science students is not optimal [24, 25]. In Ethiopia, HIV testing among adolescents and young people in general also remains low, especially among those outside health-related training environments, suggesting that being a student does not automatically translate into better testing practices. At the same time, medical and health science students are future healthcare providers, meaning their attitudes and behaviors can strongly influence how communities perceive and use HIV testing services. For this reason, they remain an important group for targeted efforts to improve early HIV testing and strengthen wider HIV prevention strategies [26].

Though numerous studies in Sub-Saharan Africa have assessed the awareness and acceptability of HIV self-testing [27, 28], there is very limited evidence available on the practice and predictors of HIV self-testing among governmental medical college students in Ethiopia [15, 29]. Most of the studies in Ethiopia have been conducted among key populations like female sex workers, but not among medical college students as a separate group [15]. The magnitude of HIV self-testing practice and factors associated with it among medical college students is crucial for the development of interventions and improvement of the national HIV prevention program [22].

Therefore, this study aimed to determine the prevalence of HIV self-testing practice and identify socio-demographic, behavioral, and knowledge-related factors associated with its utilization among governmental medical college students in Addis Ababa, Ethiopia, in 2024, in order to generate evidence that can inform targeted interventions and strengthen campus-based HIV prevention strategies.

## Methods

### Study area and period

The study was conducted in government medical and health science colleges in Addis Ababa, the capital of Ethiopia. There are five government medical and health science colleges in Addis Ababa. These colleges are Tikur Anbessa Specialized Referral and Teaching Hospital, Saint Paul’s Specialized Hospital, Zewditu Memorial Hospital, Menelik II Medical and Health Science College, and Sefere Selam. These colleges offer undergraduate training programs in medicine, nursing, midwifery, pharmacy, laboratory sciences, and other health-related fields [30]. Collectively, these institutions have an estimated total student of approximately 6,859 medical and health science students across all programs. Data for the study were collected from May 5 to June 5, 2024.

### Study design

An institution-based cross-sectional analytical study design was employed.

### Population

#### Study population

All eligible adult (≥ 18 years) medical students enrolled at governmental medical and health science colleges in Addis Ababa during the study period.

#### Inclusion and exclusion criteria

##### Inclusion criteria

Students aged 18 years or older who were officially enrolled in the 2023/2024 academic year at the selected governmental medical and health science colleges in Addis Ababa.

##### Exclusion criteria

Medical students who were seriously ill requiring hospital admission during the data collection period were excluded.

#### Sample size and sampling technique

##### Sample size determination

The sample size for this study was calculated based on the prevalence of awareness and willingness to perform oral HIV self-testing among health science students, which was estimated to be 67% in a previous study [31]. Using a 95% confidence level, a 5% margin of error, and a 10% allowance for non-response, the final sample size was calculated to be 375 students.

For the second objective, the sample size was calculated using Epi Info version 7 to assess factors associated with HIV self-testing practice. The calculation assumed a 95% confidence level and 80% statistical power. The outcome variable was HIV self-testing practice, and several potential associated factors were considered, including recent HIV testing history, family history of HIV, and history of sexually transmitted infections (Table 1). The required sample sizes for these factors ranged from 208 to 240.

Since the sample size calculated for the first objective was larger, it was used as the final sample size to ensure adequate statistical power for both the descriptive and analytical objectives of the study.

**HIV self-testing practice and associated factors are presented in** Table 1.

**Table 1:** HIV self-testing practice and associated factors among governmental medical college students in Addis Ababa, Ethiopia, 2024

Variables	% (Yes)	% (No)	CI	Power	Total sample size
recently tested for HIV	74.2	25.8	95%	80%	228
Family history of HIV	72.8	27.32	95%	80%	240
History of STI	67.9	32.1	95%	80%	208

##### Sampling techniques

In Addis Ababa, there are five governmental medical and health science colleges. Two of these colleges, Tikur Anbessa Specialized Referral and Teaching Hospital and Saint Paul’s Specialized Hospital, were selected using a simple random sampling technique. The total number of students in selected colleges constituted the sampling frame (*N* = 3,342).

Based on the number of students enrolled in each college prior to the study period, the calculated sample size was distributed proportionally to each institution. The sampling frame was constructed using the total number of students in the selected colleges, and a systematic sampling technique was employed to select participants. A lottery method was used to determine the starting point for each interview, and subsequent participants were selected at regular K-intervals, calculated as N/*n* = 3,342/375 = 8.9 ≈ 9, until the required sample size was achieved.

##### Data collection procedures

Data collection was carried out from May 5th to June 5th, 2024, using a structured self-administered questionnaire adapted from a study done in Northwest Ethiopia [32], with some modifications. The questionnaire consisted of questions related to socio-demographic factors, factors related to HIV testing, behavioral factors, and health-seeking behavior. Awareness of HIV self-testing was measured by asking the respondents, “Have you ever heard of an oral HIV self-test?” (Yes = 1, No = 0) after a brief description of the process. HIV self-testing behavior was measured by asking the respondent if they had ever done an HIV self-test (Yes = 1, No = 0). Respondents who had self-tested were asked to report their latest test outcome, which was classified as reactive (presence of both control and test lines) or non-reactive (presence of control line only), as per the manufacturer’s guidelines.

The purpose of the study was explained to participants before data collection, and their verbal permission was obtained. The participation was voluntary, and they could withdraw themselves from it at any stage they desired.

Data collection was done by trained B.Sc. nurses who were standardized on the objectives of the study, ethics, confidentiality, and interviewing. To ensure the quality of the data collected, the questionnaire was carefully designed in English. The questionnaires were reviewed daily by the principal investigator for completeness and consistency, and corrections were made immediately for any inconsistencies. To reduce social desirability bias, the interviews were done in private rooms, and the participants were assured of confidentiality and anonymity, and that the data would be used only for research purposes without any personal identifiers.

Pilot testing was done on 10% of the sample at Adama Hospital & Medical College, which is not part of the main study, to check for clarity, completeness, content validity, and reliability. Modifications were done based on the pilot test results.

In order to reduce the non-response rate, interview appointments were made at a convenient time, while follow-up interviews were conducted if respondents failed to attend. Students who refused to participate in the study were categorized as non-responsive and no replacements were assigned for them. The non-response rate was considered to be 10%.

### Study variables

#### Dependent variables

##### HIV self-test practice (Yes/No)

Responses were categorized as “Yes” for students who had performed the HIV self-test at least once and “No” for those who had never undertaken the test. There was no laboratory confirmation or medical record to validate the results; they relied entirely on self-reporting. This measure captures lifetime self-reported usage and was not independently verified.

#### Independent variables

##### Socio-demographic factors

The independent variables include age, sex, academic level, marital status, academic year, source of income, residence, and department.

##### Knowledge-related factors

Knowledge-related factors include HIV self-testing and history of occupational exposure.

##### Behavioral factors

It includes ever having had sex, age of first sex, number of sexual partners, ever having had an STI, Frequent condom use, drug use, alcohol use, and Perceived risk of HIV infection.

### Data analysis procedures

The completed questionnaires were reviewed for completeness and consistency by the supervisors, and errors that were detected were corrected immediately. The data was entered by professional data entry clerks using EpiData version 3.1 and then exported to SPSS version 26 for analysis. Before analysis, data cleaning was done using frequency and cross-tabulation to detect missing and inconsistent values.

Bivariable logistic regression analysis was initially conducted to assess the crude association between each independent variable and HIV self-testing practice. Variables with p-values < 0.25 in the bivariable analysis, together with variables supported by previous literature, were included in the multivariable logistic regression model to minimize the exclusion of potential predictors.

The final multivariable logistic regression model was adjusted for socio-demographic, behavioral, HIV-related, and health-seeking factors. The potential presence of confounding was assessed by examining changes in effect estimates during model building. Multicollinearity was evaluated using the Variance Inflation Factor (VIF), and all variables had VIF < 10, indicating no significant multicollinearity.

Both crude odds ratios (COR) and adjusted odds ratios (AOR) with 95% confidence intervals (CI) were reported. Statistical significance was declared at *p* < 0.05.

### Ethical consideration

Ethical approval for this study was obtained from the Yanet College Ethics and Research Committee, and permission was obtained from the Addis Ababa Public Health Research and Emergency Management Directorate (details are provided in the Declaration section).

## Results

### Socio-demographic characteristics

A total of 375 students took part in the study, giving a response rate of 100%. The method of sampling and the actual number of participants included in the analysis are shown in.

**Fig. 1:**
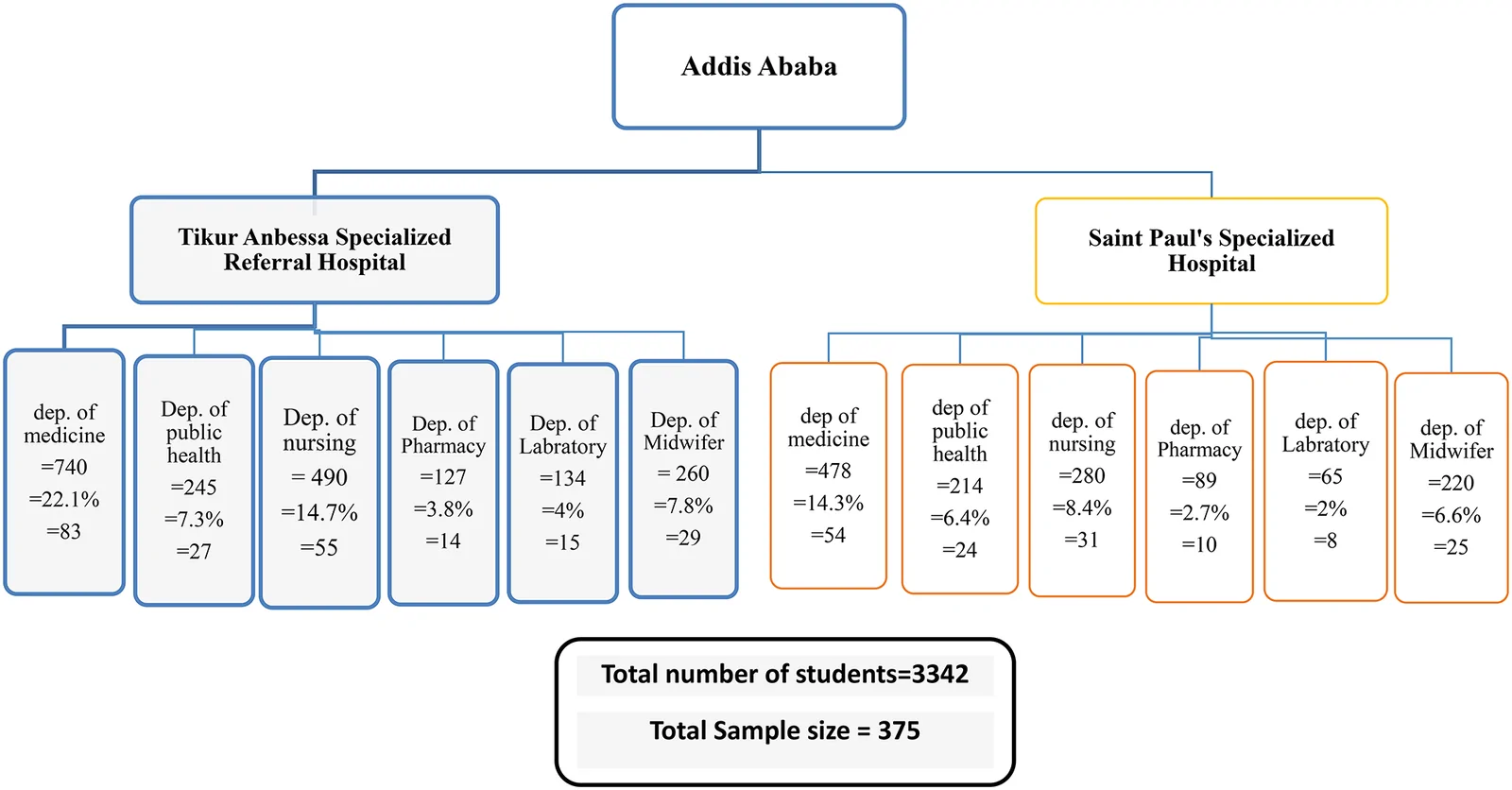
Schematic presentation of sampling procedure of HIV Self-Testing Practice and Associated Factors among Governmental Medical College Students in Addis Ababa, Ethiopia, 2024

**The sampling procedure is presented in** Fig. 1.

Most of the respondents were younger than 25 years old 78.1% (*n* = 293). Males dominated the study at 60% (*n* = 225), while females comprised 40%(*n* = 150). Most of the respondents were taking the Medicine course 36.3% (*n* = 136), followed by nursing 22.9% (*n* = 86) and Midwifery 13.2% (*n* = 54). Almost half of the respondents were second-year students 48.9% (*n* = 183), followed by third year 27% (*n* = 101). Most of the respondents were not living with their spouse or partner 79.7%(*n* = 299), while 20.3% (*n* = 76) were living with their spouse or partner.

**“The socio-demographic characteristics of study participants are presented in** Table 2.**”**

**Table 2:** Socio-demographic characteristics of study participants based on stratified sampling from Saint Paul’s Specialized Hospital and Tikur Anbessa Specialized Referral Hospital in Addis Ababa, Ethiopia, 2024

Variables	Category	Frequency (*n*)	(%)
Age	< 25	293	78.1
	≥ 25	82	21.9
Sex of respondent	Female	150	40
	Male	225	60
Residence	Rural	197	52.5
	Urban	178	47.5
Departments	Medicine	136	36.3
	public health	52	13.9
	Nursing	86	22.9
	Pharmacy	24	6.4
	Midwifery	54	14.4
	Laboratory	23	6.1
Year of study	2nd	183	48.9
	3rd	101	27.0
	4th	78	20.8
	5th	13	3.2
Currently living with your spouse/partner	Yes	76	20.3
	No	299	79.7

### Awareness of HIV self-testing practice

HIV self-testing (HIVST) awareness was measured using a self-reported method where participants were asked if they had ever heard of an oral HIV self-test (Yes/No). This indicator captures the participants’ ability to recall their exposure to information about HIVST, as opposed to knowledge.

A total of 57.6% (*n* = 216) of the respondents reported having heard of oral HIV self-testing, while 42.4% (*n* = 159) reported that they had not heard of it before.

**Fig. 2:**
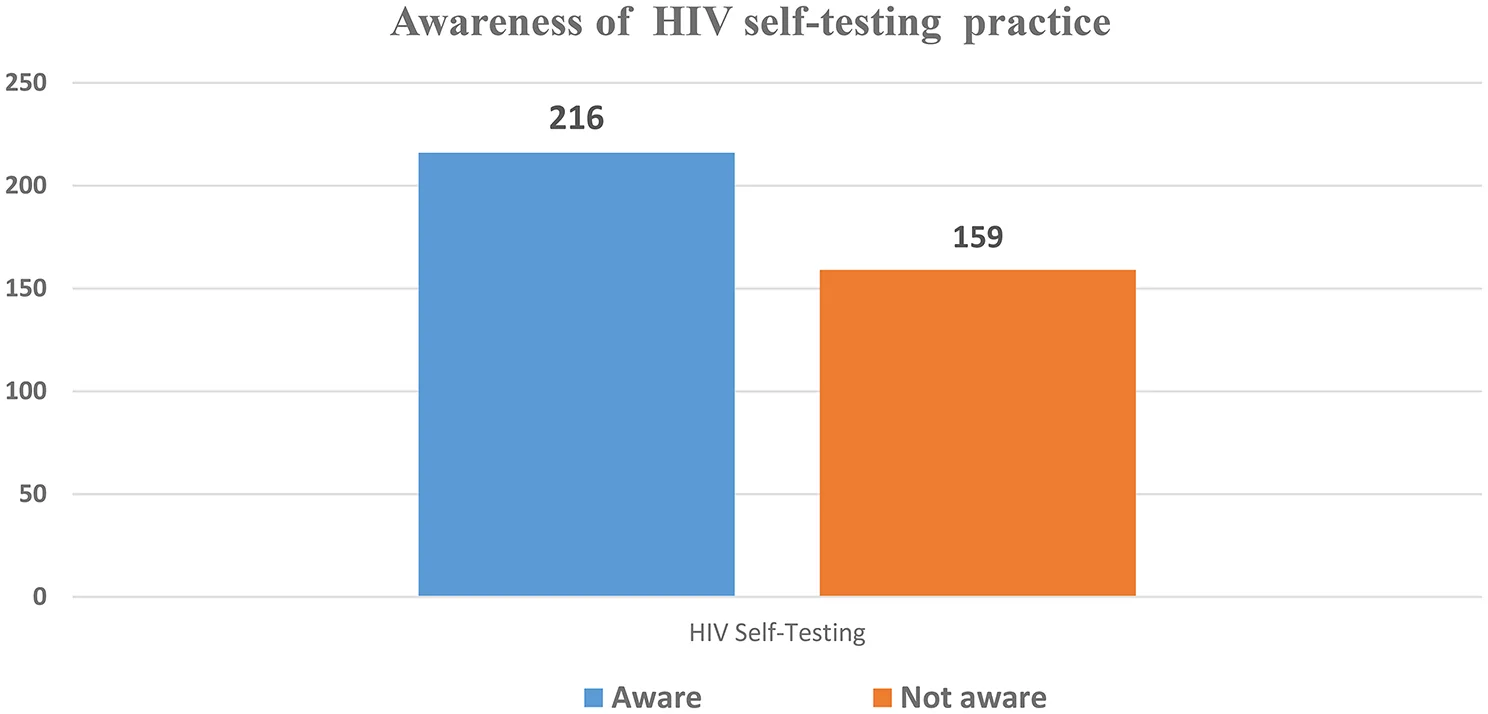
Awareness of HIV self-testing practice among governmental medical college students in Addis Ababa, Ethiopia, 2024

**“Awareness of HIV self-testing practice is shown in** Fig. 2.**”**

#### Behavioral characteristics of HIV self-testing practice

The measurement of behavioral characteristics was done using self-reported data from structured questionnaire items. These characteristics are based on the participants’ self-reported behavior and perceptions at the time of the survey and were not independently validated.

Regarding sexual behavior, 71.7% (*n* = 269) reported sexual intercourse within the past three months. Among them, 79.7% (*n* = 299) had one sexual partner, while 20.3% (*n* = 76) had three or more partners. The majority, 69.1% (*n* = 259), reported not using condoms consistently.

In terms of sexual debut, 70.4% (*n* = 264) initiated sexual intercourse at 19 years or older, while 29.6% (*n* = 111) did so at 18 years or younger. Only 13.9% (*n* = 52) reported a history of STI.

Alcohol use was reported by 16.5% (*n* = 62), recent hospital visits by 28.5% (*n* = 107), and recent HIV testing by 14.9% (*n* = 56). Perceived susceptibility to HIV infection was low, with 9.1% (*n* = 34) reporting perceived risk.

**“The behavioral characteristics of HIV self-testing practice among governmental medical college students are presented in** Table 3.**”**

**Table 3:** Behavioral characteristics of HIV self-test practice among governmental medical college student in Addis Ababa. Ethiopia 2024

Variables	Category	Frequency (*n*)	%
Have you ever tested for HIV?	Yes	207	55.2
	No	168	44.8
Have you ever been diagnosed with STI	Yes	52	13.9
	No	323	86.1
Have sex within the last 3 months?	Yes	269	71.7
	No	106	28.3
Do you use condoms consistently	Yes	116	30.9
	No	259	69.1
Have you ever drunk alcohol?	Yes	62	16.5
	No	313	83.5
Do you visit hospital last 3 months?	Yes	107	28.5
	No	268	71.5
Do you have ordinary check up	Yes	40	10.5
	No	335	89.5
Chronic disease	Yes	31	8.3
	No	344	91.7
Family history of HIV	Yes	21	5.6
	No	354	94.4
Do you take recent HIV related Training	Yes	37	9.9
	No	338	90.1
Do you test HIV recently	Yes	56	14.9
	No	319	85.1

#### Practice of HIV self-testing

The experience with HIV self-testing was determined using a single self-administered question asking participants if they had ever used an HIV self-test (Yes/No). This outcome variable is a lifetime self-reported measure and has not been independently validated using medical records.

A total of 19.2% (*n* = 72) of participants reported that they had ever used an HIV self-test, while 80.8% (*n* = 303) reported that they had never used an HIV self-test. This proportion is a self-reported lifetime experience of HIV self-testing and not actual clinical practice or experience in the past specified time period.

**Fig. 3:**
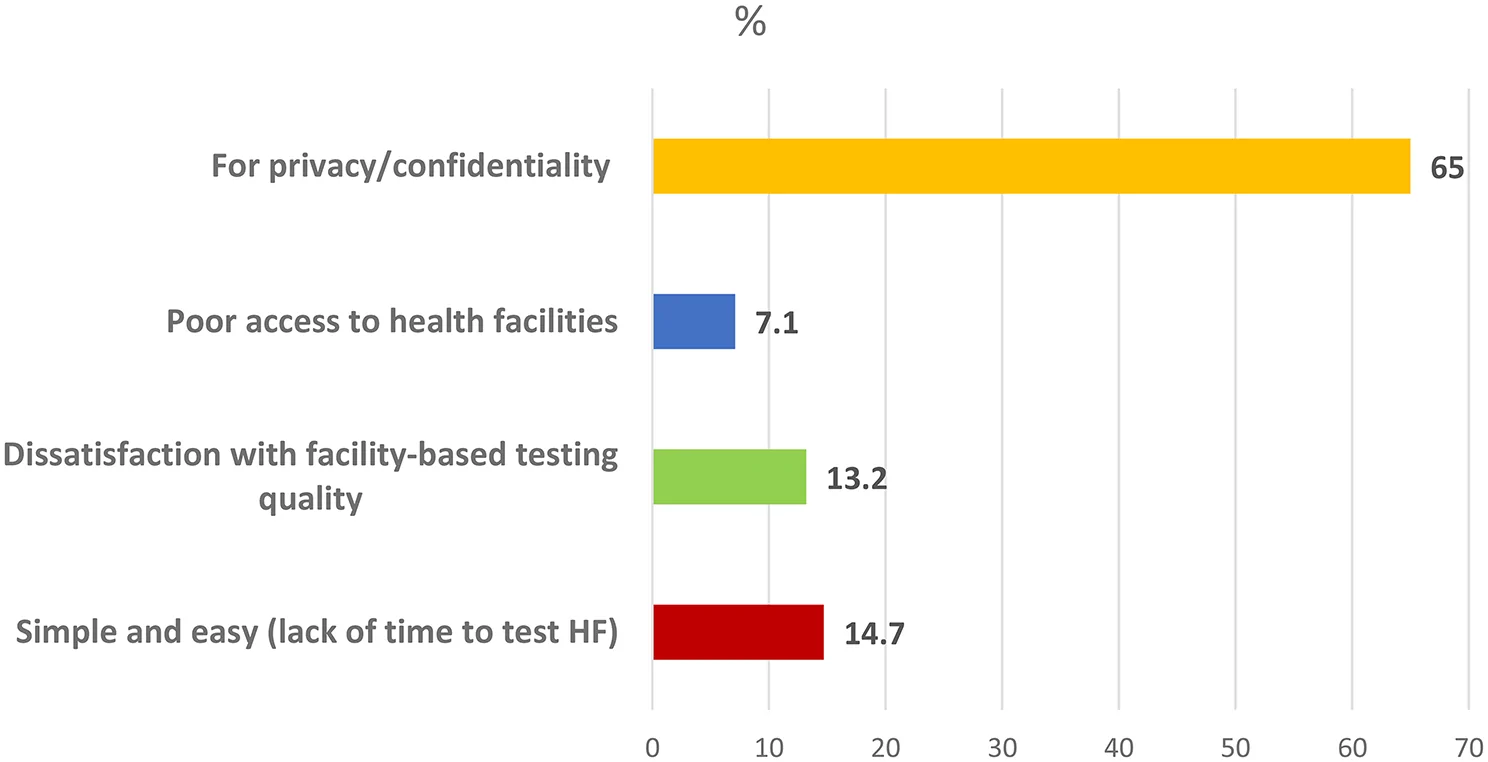
Reasons for HIV Self-test practice among governmental medical college student in Addis Ababa. Ethiopia 2024

**“The reasons for HIV self-testing practice are shown in** Fig. 3.**”**

#### Factors associated with HIV self-testing practice

In the multivariable analysis, several factors were significantly associated with the practice of HIV self-testing among students. Students who had participated in HIV program had 3.27 times higher odds of HIV self-testing compared to those who had not participated (AOR = 3.27; 95% CI: 1.30–8.21). Male students had 1.74 times higher odds of reporting HIV self-testing compared to female students (AOR = 1.74; 95% CI: 1.02–2.82). Students who had experienced recent HIV risk were 2.84 times higher odds of using an oral HIV self-test compared to those without recent risk (AOR = 2.84; 95% CI: 1.35–5.98). Furthermore, students with a history of STI diagnosis were 3.18 times higher odds of reporting HIV self-testing compared to those without an STI history (AOR = 3.18; 95% CI: 1.42–7.12).

**“The factors associated with HIV self-testing practice are presented in** Table 4.**”**

**Table 4:** Multivariable analysis for factors associated with practice of HIV self-testing among governmental medical college student in Addis Ababa, Ethiopia, 2024

Variables	HIV Self-test practice		Unadjusted and adjusted OR 95% Confidence Interval	
	No	Yes	COR (95%CI)	AOR (95%CI)
Age (years)				
< 25 ≥ 25	250 (78.9) 67 (21.1)	43 (79.1) 15 (20.9)	1 0.66 (0.39–1.11)	1 0.54 (0.29–1.01)
Sex of respondent				
Female Male	137 (43.2) 180 (56.9)	13 (22.5) 45 (77.5)	1 2.00 (1.28–3.13)	1 1.74 (1.02–2.82) *
Residence				
Rural Urban	164 (51.74) 153 (48.5)	33 (60.3) 25 (39.7)	1 0.64 (0.35–1.18)	1 0.64 (0.32–1.27)
Year of study				
2nd 3rd 4th 5th	155 (48.90) 85 (26.8) 66 (20.82) 11 (3.47)	26 (49.1) 20 (30.3) 11(18.75) 1 (1.9)	1 1.44 (0.84–2.48) 0.88 (0.50–1.55) 2.54 (0.53–11.9)	1 1.26 (0.69–2.27) 0.75 (0.40–1.41) 1.64 (0.31–8.74)
Currently living with your spouse/partner?				
No Yes	246 (77.60) 71 (22.40)	48 (90.6) 5 (9.4)	1 0.36 (0.13–0.94)	1 0.37 (0.13–1.04)
Have you ever been diagnosed with STI				
No Yes	274 (86.44) 43 (13.6)	49 (85.0) 9 (15.0)	1 2.41 (1.19–4.88)	1 3.18 (1.42–7.12) *
Have sex within the last 3 months?				
No Yes	231 (72.9) 86 (27.3)	38 (64.3) 20 (35.7)	1 1.62 (0.88–2.99)	1 1.25 (0.60–2.62)
Do you take recent HIV related training				
No Yes	295 (93.1) 22 (6.9)	43 (79.1) 15 (20.9)	1 3.11 (1.38–7.03)	1 3.27 (1.30–8.21) *
Have you ever drunk alcohol?				
No Yes	267 (84.2) 50 (15.8)	46 (79.4) 12 (20.6)	1 1.55 (0.82–2.90)	1 1.77 (0.90–3.48)
Recent HIV risk exposure				
No Yes	107 (91.5) 10 (8.5)	234(90.5) 24 (9.5)	1 2.15 (1.10–2.40)	1 2.84 (1.35–5.98) *
Chronic disease				
No Yes	298 (94.0) 19 (6.0)	46 (84.8) 12 (15.2)	1 2.38 (0.95–5.99)	1 2.06 (0.68–6.28)
Family history of HIV				
No Yes	306 (96.5) 11 (3.5)	48 (89.6) 10 (11.4)	1 2.89 (0.96–8.70)	1 3.44 (0.87–13.57)

* Statistically significant at p-value < 0.05

Note: Percentages are column percentages based on HIV self-test practice

## Discussion

In this study, the proportion of students who have ever used oral HIV self-testing was 19.2% (*n* = 72). This finding is consistent with studies conducted in Kenya, Rwanda, Tanzania, and Zimbabwe, which reported that the prevalence of HIV self-testing among young adults and university students ranged between 15% and 25% [14, 30, 33, 34]. These similar prevalence rates may be due to similar levels of awareness, barriers to access, and the novelty of HIV self-testing programs in Eastern and Southern Africa.

There are several studies that have identified the key factors that hinder the use of HIV self-testing, including a lack of awareness about the availability and accuracy of HIV self-tests, lack of access to testing services in rural and campus settings, inconsistent supply and distribution of test kits, regulatory and policy challenges that hinder the widespread implementation of HIV self-testing, and concerns about the accuracy and interpretation of test results in the absence of healthcare provider support [15, 21, 22, 35]. Despite these challenges, oral HIV self-testing is generally preferred by many people because it provides confidentiality, privacy, and convenience, allowing people to test in the privacy of their homes without fear of stigma and discrimination [18, 19, 31].

Involvement in HIV-related programs was significantly associated with increased odds of HIV self-testing practice. Students involved in HIV-related programs had 3.27 times higher odds compared to those who did not participate. This result is supported by studies conducted in Kenya, which indicated that students who were exposed to HIV-related educational programs had increased awareness and knowledge of HIV prevention strategies [28, 36]. HIV programs offer students with opportunities for education, training, and resource access, which may reflect increased exposure to HIV-related information and awareness of self-testing. Furthermore, Students who had recently experienced HIV-related risk had higher odds of HIV self-testing practice, indicating that students who perceive higher risks are more proactive in seeking information and testing [37].

Male students had 1.74 times higher odds of performing oral HIV self-testing (HIVST) than female students. This result is consistent with research that took place in Uganda, Zimbabwe, and other sub-Saharan African countries, where males were more likely to use HIVST [13, 35, 38]. One of the possible reasons for this result is that male students may feel more embarrassed when accessing conventional health facilities for HIV testing, and therefore the confidentiality and privacy offered by oral HIVST are more attractive [13, 20]. On the other hand, some research that took place in Kenya [28], has found that female students were more likely to use HIVST, indicating that gender differences in testing behaviors may be affected by cultural factors, social norms, and expectations of perceived HIV risk or health-seeking behaviors.

Students who had recent HIV risk exposure had 2.84 times higher odds of practicing oral HIV self-testing (HIVST) than those who had not been exposed to such risks. This result is supported by a study conducted in Rwanda, which also found that those who had recently been exposed to risks were more likely to use HIVST [39]. The relationship between the variables may be explained by health-seeking behavior, where students who feel they are at a higher risk of HIV infection are more encouraged to seek testing to determine their status early. Oral HIVST is ideal for such groups of people since it provides confidentiality, convenience, and simplicity, which enables the individual to conduct the test in private without the inconveniences that come with conducting HIV counseling and testing in facilities, such as stigma, waiting in line for long, or when the facility is closed [35, 40].

In this study, students who reported a history of sexually transmitted infection (STI) had 3.18 times higher odds of performing oral HIV self-testing (HIVST) compared to those who had not been previously diagnosed with an STI. This is a very strong association that is supported by the fact that people who have had previous experiences with STIs have been shown to be more likely to test for HIV and other protective behaviors because of increased risk perception and health-seeking behavior. Studies conducted among university students in Uganda revealed that having had an STI and other sexual risk exposures was a determinant of willingness to self-test for HIV, indicating that people who have had previous experiences with STIs feel more vulnerable and are motivated to test for HIV when the opportunity arises [38]. Individuals who reported a history of sexually transmitted infection (STI) tend to identify themselves as being at a higher risk for HIV infection because STIs are a biological and behavioral factor that increases vulnerability to HIV infection. Studies conducted among adolescents and adults have found that individuals who reported a history of STI or prior STI testing are more likely to access HIV testing services than those who do not have such a history [41].

In conclusion, these results show that both individual risk perception and programmatic exposure are essential for HIVST awareness and utilization. Interventions to increase HIVST use should be directed at increasing HIV education programs and making HIVST kits widely available, in addition to addressing structural barriers that impede access to private and convenient testing. Targeted communication to at-risk groups and those with a history of STIs would likely further improve HIVST use among students.

### Limitation

There are also some limitations in this study. HIV self-testing and other variables were assessed based on using self-reporting data with lifetime recall, which is prone to social desirability bias and can result in overestimating HIV self-testing rates and other socially desirable behaviors. Recall bias could also play a role as the participants would find it difficult to recall their past and lifetime experiences. Furthermore, self-reporting was used to collect information about HIV self-testing and STI history without any medical records or laboratory confirmation. The cross-sectional study design also has limitations in establishing causality. Lastly, since this study was conducted among governmental medical college students in Addis Ababa, who are likely to have better health literacy and access to information, the results might overestimate the level of awareness and self-reported HIV self-testing compared to the general population or vulnerable groups in Ethiopia.

## Conclusion and recommendations

The uptake of oral HIV self-testing among students of government medical and health science colleges in Addis Ababa has been reported as being quite low (19.2%), which poses a potential public health threat, consistent with reports from related research conducted in similar situations across other parts of sub-Saharan Africa including Ethiopia. Self-testing was reported to be more common among male students, students who had participated in HIV programs, and those who had previously engaged in risky sexual behavior or had sexually transmitted infections, implying that perception of risk and exposure to HIV information is associated with self-testing behavior.

Improved access to targeted HIV education and information programs and improved access and affordability of self-testing kits is required. Future studies using longitudinal or mixed-methods approaches are recommended to investigate factors associated with HIV self-testing among students.

## Data Availability

The datasets used and/or analyzed during the current study are available from the corresponding author on reasonable request.

## Abbreviations

AIDS: Acquired Immunodeficiency Syndrome
CIT: Client Initiated Testing and Counseling
HAART: Highly Active Antiretroviral Therapy
HTC: HIV Testing and Counseling
AIS: AIDS Indicator Survey
NACC: National AIDS Control Council
NASCOP: National AIDS and STD Control Program
PITC: Provider Initiated Testing and Counseling
PEP: Post-exposure prophylaxis
SPSS: Statistical Package for the Social Sciences
STI: Sexually Transmitted Infections
VCT: Voluntary Counseling and Testing
WHO: World Health Organization
